# Cilia, Alström Syndrome – molecular mechanisms and therapeutic perspectives

**Published:** 2008-08-15

**Authors:** Maria Mihai Cristina, Catrinoiu Doina, Marshall Jan, Stoicescu Ramona, Tiberiu Tofolean Ioan

**Affiliations:** * Department of Surgery from Medical School of the Federal University of Minas Gerais; ** Department of Surgery from Medical School of the Federal University of Minas Gerais; *** Department of Surgery from Medical School of the Federal University of Minas Gerais; **** Department of Surgery from Medical School of the Federal University of Minas Gerais; ***** Department of Surgery from Medical School of the Federal University of Minas Gerais

**Keywords:** Cilia, Alström syndrome, obesity, diabetes

## Abstract

Over the past ten years, several studies demonstrated the connections between cilia, basal bodies and human diseases with a wide phenotypic spectrum, including randomization of body symmetry, obesity, cystic kidney diseases and retinal degeneration. Alström syndrome (OMIM 203800) first described in 1959, is a rare autosomal recessive disorder caused by mutations in a novel gene of unknown function, ALMS1, located on the short arm of chromosome 2. Central features of Alström syndrome include obesity, insulin resistance, and type 2 diabetes. About 500 individuals with Alström syndrome are known worldwide. ALMS1 is widely expressed and localizes to centrosomes and to the base of cilia. We discuss the possible molecular mechanisms, clinical features, and future therapeutic options in a patient diagnosed with this rare disease.

Monogenic defects causing human obesity actually disrupt hypothalamic pathways with a profound effect on satiety and food intake. A potential contributor to obesity- cilia with impaired function or abnormal structure, creates a new link to be studied in the future, between these organelles and the genetics of obesity.

## Introduction

Cilia and flagella are slender projections from cells with a microtubule-based structure that includes a ring of nine double microtubules; each cilium grows from a basal body, a modified centrosome, a highly specialized organelle consisting of a pair of centrioles surrounded by amorphous proteinaceous matrix, the pericentriolar material [**1**]. It serves as the primary microtubule organizing center of the cell, involved in nucleation, anchoring, and release of microtubules during cell division and chromosome segregation [**2**]. Cilia protrude into the extracellular space, acting as antennae to receive extracellular signals [**3**]. A cilium is at least as complex an organelle as the ribosome, with hundreds of proteins dedicated to cilia organization. All cilia can now be viewed as sensory cellular antennae that coordinate a large number of cellular signaling pathways, sometimes coupling the signaling to ciliary’s motility or alternatively to cell division and differentiation [**4**-**5**].

Over the past ten years, several studies demonstrated the connections between cilia, basal bodies and human diseases, by the identification of evolutionarily conserved genes that are specifically associated with cilia and basal bodies [**2**]. A whole genome sequences comparison demonstrates that cilia were present in the eukaryotic ancestor of both plants and animals, but have been lost independently in the lineages of current organisms. Owing to the involvement of primary cilia in diverse cellular processes, mutations in centrosomal/ciliary proteins result in human disorders with a wide phenotypic spectrum, including randomization of body symmetry, obesity, cystic kidney diseases and retinal degeneration [**4**-**5**].

An increasing number of genetic diseases are associated with defects in ciliogenesis or ciliary’s function [**6**], including cystic kidney disease, infertility, retinal degeneration, hydrocephalus, laterality defects and chronic respiratory problems, Bardet-Biedl, Alström, Orofaciodigital and Meckel syndromes [**7**-**9**]. Particularly, the proteins associated with diseases that are phenotypically similar, such as BBS and ALMS, also localize to the centrosomal regions and within the basal bodies of ciliated cells, and studies of mouse models for BBS (*Bbs1–/–, Bbs2–/–, Bbs4–/– and MKKS–/–*) have implicated several BBS proteins in ciliary’s function and intracellular trafficking. Most of the gene products implicated in the ciliopathies affect ciliary’s function (e.g. motility or signaling) rather than cilium formation [**8**, **10**, **11**].

Alström syndrome (OMIM 203800) was first described in 1959 [**12**]; it is a rare autosomal recessive disorder caused by mutations in a novel gene of unknown function, *ALMS1*, located on the short arm of chromosome 2. Central features of Alström syndrome include obesity, insulin resistance, and type 2 diabetes [**13**].

**Figure:**
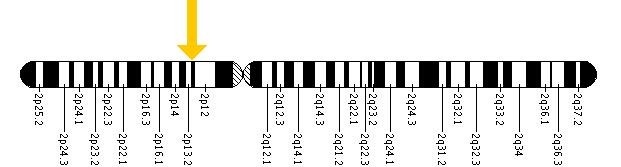
Cytogenetic Location: 2p13 Molecular Location on chromosome 2: base pairs 73,466,393 to 73,690,553 (http://ghr.nlm.nih.gov/gene=alms1)

Two independent groups (Collin et al., 2002; Hearn et al., 2002) reported that mutations in human *Alms1* are responsible for this syndrome in humans. *Alms1* is a widely expressed gene that encodes a 461kDa [4169 amino acid (a.a.)] protein with a largely unknown function. It contains a large tandem repeat domain coded by a single exon 8 comprising 34 imperfect repeats of a 44a.a. sequence. This domain constitutes 40% of the protein. ALMS1 also has a short polyglutamine segment (17 Glu repeats, a.a. 13–29), which is followed by seven alanine residues (a.a. 30–36). In addition to a predicted leucine zipper domain (a.a. 2480–2501), this protein has an evolutionary conserved motif, the Alms motif, near the C terminus [**14**-**16**]

*ALMS1* is comprised of 23 exons encoding a protein of 4169 amino acids. The ALMS1 protein is ubiquitously expressed and localizes to centrosomes and basal bodies of ciliated cells, perhaps playing an important role in cilia function and intraflagellar transport. To date, the mutations reported in *ALMS1* have been nonsense and frameshift variations (insertions or deletions) and one reciprocal translocation that are predicted to cause premature protein truncation. In total, 79 disease-causing variants have been reported.

ALMS1 is widely expressed and localizes to centrosomes and to the base of cilia. In fibroblasts with disrupted ALMS1, primary cilia and the microtubule cytoskeleton appear to be normal, suggesting that the ALMS phenotype results from impaired ciliary’s function rather than from abnormal ciliary’s structure (Hearn et al., 2005) [**16**].

The central role for basal body and centrosome dysfunction in the pathogenesis of obesity, insulin resistance, and type 2 diabetes was revealed by several studies about the complex phenotype of Bardet-Biedl syndrome [**17**]. Also, Collin et al. (2005) [**18**] reasoned that the infantile obesity observed in individuals with syndrome is probably caused by mutation of ALMS1, as it constitutes a relatively early (as early as 6 months) phenotype observed in all affected children. The early onset of obesity, anecdotal reports of hyperphagia, and the sensory deficits observed in individuals with syndrome suggested to Collin et al. (2002) [**14**] that the obesity is due to loss of ALMS1 function in the central nervous system. Nearly all individuals with syndrome develop type 2 diabetes (125853), suggesting that ALMS1 may be involved in 'diabesity,' a term used by Collin et al. (2002) [**14**] for combined obesity and diabetes susceptibility due to altered function of a single gene. This distinguishes it from the common forms of obesity, in which the genes that are presumably involved appear to interact with independently segregating genes that confer diabetes susceptibility, as not all of obese individuals develop type 2 diabetes.

## Case report

The patient (A.U.), is a 21-year-old daughter of a nonconsanguineous parents, has no siblings and not known affected relatives. She was born normally, birth at weight was 2.9kg. During her first year of life were noted increased weight gain and progressive and worsening pathological ocular signs: nystagmus, convergent strabismus, photophobia (at 15 months), reduced visual acuity. The first sign, nystagmus, was noted at 4 months of age, then she developed progressive retinal distrophy and blindness by 10 years of age. Infantile obesity (16 kg at 11 months of age) was also noted and progressed with age. Sensorineural hearing impairment was noted at 10 years of age and type 2 diabetes mellitus was diagnosed at 12 years of age.

In her evolution were noted: abnormal liver function tests, reccurent urinary tract infections, incontinence, scoliosis, kyphosis, acanthosis nigricans, obesity (123kg/167cm, BMI = 44.1kg/m2) but, with normal intelligence, normal extremities, normal secondary sexual characteristics. No menstruation, chronic obstuctive pulmonary disease (abnormal spirometry), gastro-esophageal reflux disease (pH-manometry).

The initial diagnosis was Bardet-Biedl syndrome, based on severe retinal degeneration, with secondary blindness, hearing impairment, obesity, diabetes mellitus (insulin resistant) and several associated metabolic disturbances.

In infancy, due to obesity, she was evaluated for thyroid and metabolic dysfunctions and were excluded alterations of glucose metabolism, thyroid functionality, lysosomal storage diseases and aminoacidopathies. The electroretinogram (ERG), performed at 1 year of age diagnosed *retinitis pigmentosa*. The ophtalmologic evaluation showed posterior bilateral cataracts, a very pale papilla with clear-cut borders, diffuse retinal dystrophy with dispersed pigment accumulations and bilateral pigmented macular scarring. The audiometric examination showed bilateral symmetric hypoacusia.

In evolution, several laboratory tests evidenced hyperglycemia, hypertriglyceridemia, hypercholesterolemia and increased liver transaminases. In the following years, there was a progressive worsening of the metabolic function. Non-insulin dependent diabetes mellitus with hypertriglyceridemia developed and a treatment with diet and lipid lowering drugs was started. Hyperglycemia resistant to diet therapy and acanthosis nigricans on her neck occurred and she was advised to start therapy with metformin.

Abdominal ultrasound showed hepatomegaly and an echostructure compatible with steatosis.

Pituitary-thyroid axis: basal thyroid hormones and TRH stimulated pituitary function (normal TSH, fT3, fT4; negative search for anti-thyroid auto-antibodies) showed no abnormalities. Pituitary-adrenal axis: normal rhythm of cortisol and ACTH and normal values of 24h-free urinary cortisol were observed; the search of anti-adrenal auto-antibodies was negative. Pituitary-gonadal axis: pituitary gonadotrophins (LH, FSH), progesterone and estradiol measured during the follicular and luteal phases were normal while the GnRH stimulation test showed an increased and prolonged response.

Prolactin: the values for prolactin were normal before and after stimulation with TRH.

GH-IGF-I axis: during the first hospitalization in our department, at 18 years of age, we found low levels of IGF-I. Growth hormone stimulatory tests: insulin provocative test revealed a GH-deficiency.

Calcium-phosphorus metabolism: the levels of plasma and urinary calcium, phosphorus, magnesium, parathormone, and calcitonin showed no abnormalities.

The MRI study of the diencephalic and pituitary region was normal.

**Figure:**
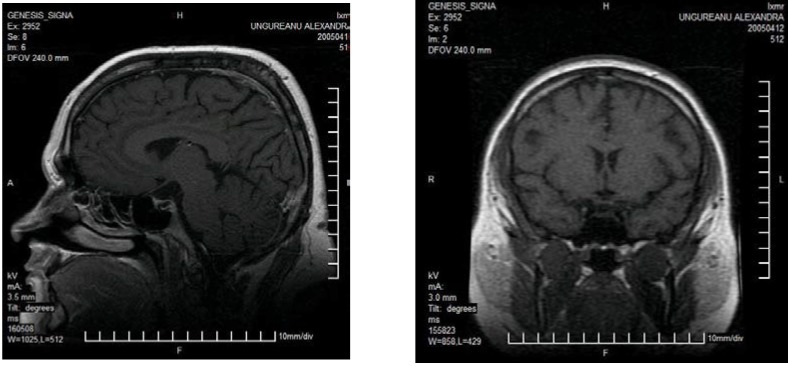
Magnetic Resonance Imaging (MRI) of the Head: normal findings

Glucose metabolism: during her first hospitalization the patient had fasting hyperglycemia in the morning (12.5mmol/L), increased levels of fructosamine (435μmol/L), HbA1c (11.9 %), basal insulin (49mU/L) and glycosuria (55.5mmol/L). Glycaemia normalized (4.3mmol/L) with the administration of Metformin. The tests for anti-islet cell antibodies (ICA) and glutamil acid anti-decarboxylase (GAD) antibodies were negative.

The genetic testing performed at Jackson Laboratory, Bar Harbor, Maine revealed in exon 16, in the ALMS1 gene in patient's DNA, 2 different mutations: 10753 C>T Ter and 10780 C>T Ter (Jackson Laboratory, Bar Harbor, Maine).

Among its diverse spectrum of clinical features are phenotypes associated with deficiencies of the GH/IGF-I axis, including short stature, obesity, insulin resistance, hypertriglyceridaemia and heart failure. Because GHD (growth hormone deficiency) and Alström Syndrome share some clinical and metabolic features, we studied the GH-IGF1 axis, using MRI techniques, dynamic tests (insulin tolerance test, ITT), MPHD (multiple pituitary hormone deficiency), and the metabolic disturbances related to GHD in this adolescent girl with Alström Syndrome. 

The subject had anthropometric and laboratory parameters checked at baseline. After a 12-hour overnight fast, blood specimens were obtained for plasma glucose, triglycerides, glycosylated hemoglobin (HbA1c), and serum total, low-density lipoprotein (LDL), and high-density lipoprotein (HDL) cholesterol. Patient, who was using insulin had her treatment stopped the day before the test, but was closely monitored for the glycemic status; an insulin tolerance test (ITT), consisting of a bolus of regular human insulin (0.1U/kg body weight equivalent to 5.22 ± 0.44 U/m2 of body surface) was performed. 

Regular (short acting) insulin was administered as an I.V. bolus at a dose of 0.1units/kg. Bedside nurse monitored the blood sugar frequently; when glucose drops below 40mg/dL, the second determination for hGH and IGF1 was performed.

In patients with diabetes on insulin consideration should be given that they may be insulin resistant. In which case, larger doses of insulin may be given. We begun with a single bolus of 0.1U/kg and then re-bolus with insulin, depending on the response to the initial dose (we either give the same dose again if there was some response but insufficient, or doubling the dose if there was only minimal response to blood glucose, or giving half the dose if the hypoglycemic response was close to the desired goal). This can be repeated several times until adequate hypoglycemia is reached. In this case, we doubled the insulin dose once (0.2 U/kg), to achive hypoglycemia.

Once the goal response of a glucose < 40mg/dL was reached, the patient was fed a meal (crackers and orange juice). Blood glucose was checked at 5', 10' and 15' minutes post feeding.

We found a severe GH deficiency, defined by a peak response to insulin-induced hypoglycemia less than 3ng/dL and IGF1 concentrations less than –2SDS.

Patient AU GH1 – 0,18ng/mL

GH2 – 0,17ng/mL (after stimulation with insulin 0,2 IU/kg/dose)

IGF-1 (somatomedin c) – 67ng/mL (116-356)

The MR imaging findings were normal.

No multiple pituitary hormone deficiency (MPHD) was found, except for severe GH deficiency.

## Discussions

Recombinant Human Growth Hormone Therapy (Re-hGH) represents a novel therapeutic option for adults with human GHD. 

We used Re-hGH in this patient for 12 consecutive months, in a dose of 0,25mg/day.

The total body fat mass decreased after one year, most significant in visceral trunk location as revealed by DEXA body composition, the bone density increased with 5% after 6 months.

**Figure:**
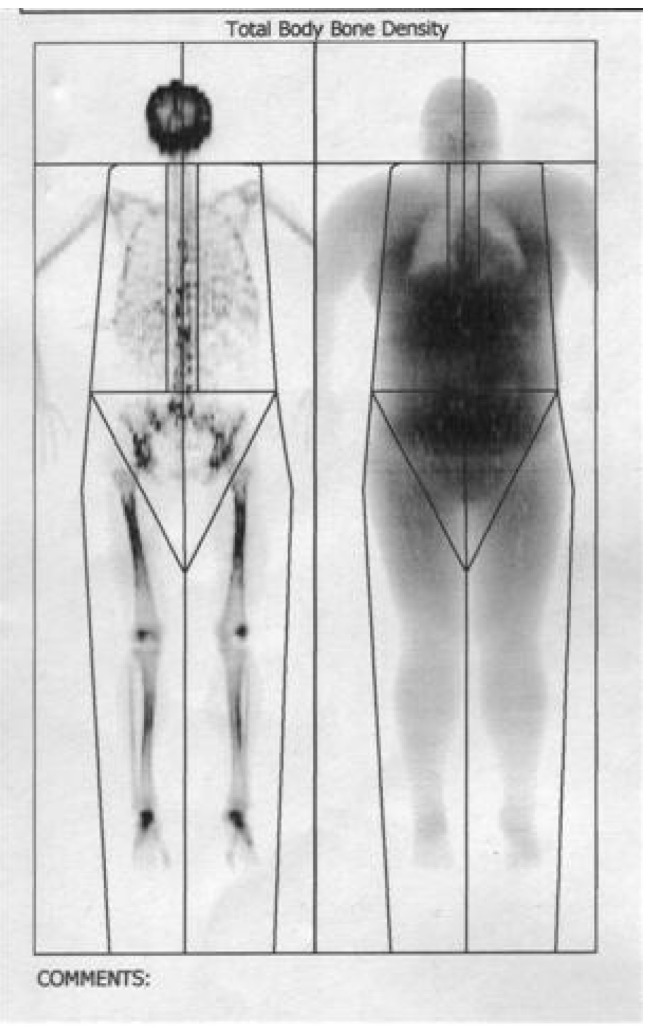
DEXA body composition analysis

***Patient:*** Alexandra UNGUREANU ***Height/Weight:*** 167cm 120kg

***Birth date:*** 25.02.1987 17,3 years ***Sex/Etnic:*** Female White

***Region*** **BMD1 (g/cm2)**

**Total 1,158**

Total body fat mass decreased after one year, most significant in visceral trunk location, the bone density increased with 5% after 6 months.

Echocardiography has shown that left ventricular mass index, fractional shortening and fiber shortening velocity improved after 12 month of low-dose therapy. No lipid metabolism improvement was noted, but a psychological well being was observed.

**Figure:**
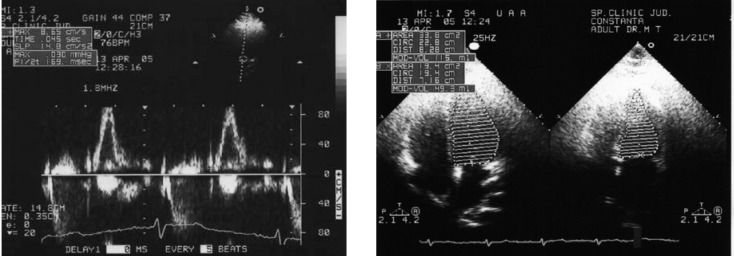
Echocardiography: left ventricular mass index, fractional shortening and fiber shortening velocity improved after 12 month of low-dose therapy with re-hGH.

The effects of circulating IGF1 on glucose metabolism are well recognized. Recent studies have demonstrated that lower baseline IGF1 levels predict the subsequent development of impaired glucose tolerance (IGT), type 2 diabetes, cardiovascular disease and a worsening state of insulin-resistance. 

Our data suggest that in adults with Alström Syndrome the GH-IGF1 axis is impaired, demonstrating a severe GH deficiency.

A defective function of ALMS1 in the target tissues of insulin action, as well as in central nervous system is probably responsible for the clinical features of Alström Syndrome.

The possibility of hypothalamic and pituitary dysfunction should always be considered in children with documented growth deceleration, particularly with known intracranial pathology (blindness, nystagmus). The child with a history of: growth failure or a documented growth deceleration, associated with: nystagmus or impaired vision and reduced serum concentrations of IGF-1 and IGFBP-3 should be considered GH deficient (or GH-inhibited), even with normal provocative tests.

Ultimately, however, the most important parameter in assessment of children with growth failure is careful clinical evaluation, including serial measurements of height and determination of height velocity.

Treatment with Re-hGH is advised in the prepuberal and puberal age in case of growth deficit and short stature, but it is still debatable if this hormone should be used in the adult subject. We believe that adult subjects with AS could benefit from adequate treatment with Re-GH in that this hormone produces favourable effects on cardiovascular and renal function (Saccà et al, 1994) [**19**]. Recent studies showed improvements in pathologies like dilative cardiomyopathy and chronic renal failure. However, the evaluation of the degree of hormonal deficiency and the starting of replacement therapy must be carried out in specialized centers since GH, which on one hand induces a lipid profile improvement, on the other hand could cause a worsening of glucose tolerance. Finally, there is the problem of establishing the effective dosage in replacement therapy that must be personalized for every single patient [**20**].

Further studies are needed to demonstrate if, Re-hGH administration in children with Alström Syndrome is beneficial, achievement of final adult height consistent with genetic potential remaining the primary therapeutic endpoint for Re-hGH therapy in the pediatric population.

In addition to its effects on bone mass, GH regulates muscle mass, muscle strength, body composition, lipid and carbohydrate metabolism, and cardiac function [**21**].

Persistent insulin resistance and dyslipidemia, together with the elevation of plasma insulin levels and lipoprotein a with GH replacement, are of concern in these patients.

Long-term follow-up data are required to assess the impact of GH replacement on cardiovascular morbidity and mortality rates in adults with GHD and AS [**22**].

Also, further studies remain to elucidate the role of hyperglycemia and hyperinsulinemia in the pathogenesis of AS. The monogenic defects cause a disruption of hypothalamic pathways the effect being on satiety and food intake, leading to obesity, insulin resistance and diabetes.

Recent evidence suggest that high glucose, advanced glycation end-products (AGE) and reactive oxygen species (ROS) act in concert to induce growth factors and cytokines that promote apoptosis and the recruitment of inflammatory cells, leading to progressive fibrosis [**23**].

The IGF factors are regarded as survival factors that display potent antiapoptotic activity, involved in all aspects of growth and development [**24**].

Interfering with IGF production, distribution or signaling may result in greater susceptibility to apoptotic stimuli [**24**].

Genetic heterogeneity is a common feature in ciliopathies, with mutant genes giving rise to differing disorders, thus making their identification and characterization all the more difficult [**25**].

The isolation of specific signaling molecules and mechanisms controlling the motility of the cilium might also result in novel therapeutic options [**26**].

Monogenic defects causing human obesity disrupt hypothalamic pathways and have a profound effect on satiety and food intake, so human obesity appears a neurobehavioral disease [**27**].

## Conclusions

The unexpected roles of cilia are suggestive for other ciliary’s functions to be discovered and to explain the broad spectrum of phenotypes observed in the ciliopathies. Cilia, the molecules and mechanisms controlling the motility of the cilium, are important tools in the pathogeny of many diseases and their study will elucidate many aspects of obesity, and diabetes seen in BBS and AS. The possibility that ciliary’s/basal body functions are involved in the regulation of glucose metabolism remain to be elucidated. Mutations in such genes determine a cellular dysfunction by disruption of cellular signaling, affecting the cellular differentiation, proliferation, migration and apoptosis. Affected cell type-specific ciliary’s signaling proteins and disruption of cellular signaling could be the major pathogenic mechanism involved. Understanding how the members of the receptor family may function in conjunction with G proteins involved in transducing of diverse signals, varying from intercellular mediators to environmental stimuli, will allow the discovering of cellular mechanisms implicated in obesity and diabetes and also will allow the development of new potential therapies.

**Abbreviations**

AGE= advanced glycation end-products

AS= Alström Syndrome

ALMS1= Alström Syndrome gene

BBS= Bardet-Biedl Syndrome

ERG= electroretinogram

GHD=growth hormone deficiency

hGH= human growth hormone

IGF1= insulin-like growth factor 1

IGFBP-3= insulin-like factor binding protein 3

ITT= insulin tolerance test

MPHD= multiple pituitary hormone deficiency

MRI= magnetic resonance imaging

Re-hGH= recombinant human hormone

ROS= reactive oxygen species
